# Safety and efficacy of sorafenib in patients with advanced thyroid carcinoma: a phase II study (NCT02084732)

**DOI:** 10.20945/2359-3997000000373

**Published:** 2021-04-27

**Authors:** Luis Felipe Fierro-Maya, Gloria Garavito González, Leonardo Javier Rojas Melo, Andrés Arturo Cuéllar Cuéllar, Alexander Carreño, Claudia Córdoba

**Affiliations:** 1 Instituto Nacional de Cancerología Unidad de Endocrinología Oncológica Bogotá Colombia Unidad de Endocrinología Oncológica, Instituto Nacional de Cancerología, Bogotá, Colombia.; 2 Instituto Nacional de Cancerología Bogotá Colombia Instituto Nacional de Cancerología, Endocrinóloga en Colsanitas, Bogotá, Colombia.; 3 Hospital Universitario San Ignacio Bogotá Colombia Servicio de Endocrinología, Hospital Universitario San Ignacio, Bogotá, Colombia.; 4 Instituto Nacional de Cancerología Unidad de Investigaciones Bogotá Colombia Unidad de Investigaciones, Instituto Nacional de Cancerología, Bogotá, Colombia.; 5 Instituto Nacional de Cancerología Unidad de Imágenes Diagnósticas Bogotá Colombia Unidad de Imágenes Diagnósticas, Instituto Nacional de Cancerología, Bogotá, Colombia.

**Keywords:** Thyroid carcinoma, metastasis, sorafenib tosylate

## Abstract

**Objective:**

Sorafenib significantly prolonged progression-free survival in patients with iodine-refractory advanced thyroid cancer. The present study was initiated before sorafenib was approved in Colombia and therefore represents an effort by an oncology institution to evaluate its efficacy and safety in this population.

**Subjects and methods:**

This phase II clinical trial had a single treatment arm. We included adult patients with progressive metastatic iodine-refractory thyroid cancer who received treatment with sorafenib 800 mg/day (400 mg every 12 hours) up to a maximum of 24 months or until the occurrence of limiting related toxicity, the progression of the disease, or voluntary withdrawal.

**Results:**

Nineteen patients received the treatment and were included in the safety analysis. However, for the efficacy analysis, 6 patients were excluded because they received only one month of therapy. Thirteen (68%) patients were women, and the mean age at diagnosis was 61.8 years. No complete responses were observed; 5 patients had a partial response (35.7%), 6 patients had stable disease, and 3 showed progression. Mean progression-free survival was calculated at 18 months (95% CI 10.7-20.3). Overall survival was estimated at 21.3 months (95% CI 17.8-24.8).

**Conclusion:**

For the first time in Colombia, the efficacy of sorafenib was evaluated in patients with advanced and progressive thyroid carcinoma refractory to radioactive iodine, with an efficacy and a safety profile similar to those previously reported.

## INTRODUCTION

Differentiated thyroid carcinoma (DTC) represents 90% of thyroid tumors, with 85% corresponding to papillar histology. Although the disease presents with a relatively indolent course, a significant percentage of patients develop locoregional relapses that can be managed by surgical resection or complementary radioactive iodine treatment. Overall survival (OS) in this setting is favorable, with 98% of patients still alive 10 years after diagnosis ( 1 ). However, approximately 7% to 23% of patients may develop distant metastases, of which up to two thirds may become refractory to radioactive iodine. The 10-year OS rate in this group is 40% to 42% ( 2 ). Patients with advanced disease who fail to respond to radioactive iodine require other therapeutic options. For this subset of individuals, and based on the knowledge of some of the genetic mutations within thyroid tumors ( 3 ), therapies inhibiting molecular targets such as tyrosine kinases have been developed. Sorafenib, a multikinase inhibitor with antiangiogenic action, was evaluated in the DECISION trial and was found to be effective in significantly prolonging progression-free survival (PFS) ( 4 ). These results, in turn, led to FDA approval for the treatment of advanced thyroid cancer in November 2013 ( 5 ). Subsequently, the use of another multikinase inhibitor, lenvatinib, was also approved in February 2015 ( 6 ), based on the results of the SELECT study, which showed significant differences compared to placebo in terms of PFS tumor response rate ( 7 ). The present study was initiated before sorafenib was approved by the regulatory entity in Colombia (INVIMA) and therefore represents an effort by an oncology institution to evaluate its efficacy and safety in a group of patients with differentiated thyroid carcinoma considered advanced, inoperable, iodine-refractory, and with metastatic involvement in progression.

## SUBJECTS AND METHODS

### Study design

This phase II clinical trial had a single treatment arm with the main objective of evaluating the efficacy and safety profile of sorafenib during a 2-year follow-up period in patients with differentiated thyroid carcinoma with inoperable locally advanced or metastatic disease involvement, refractory to radioactive iodine, in progression. The primary outcome was objective response rate according to RECIST 1.1 criteria. Disease progression (PD) was defined as a 20% increase in the sum of target lesions. A partial response (PR) was considered a decrease of ≥30% in the sum of the longest diameters of the target lesions compared to the initial value. A complete response (CR) was defined as the disappearance of all target lesions, and the disease was considered stable when the criteria for PD or PR were not met. Additional outcomes included PFS, OS, response duration, frequency and distribution of medication-related adverse effects, and the quality of life of patients at each follow-up visit. These were assessed using the Functional Assessment of Cancer Therapy, General (FACT-G) scale. This outcome is under analysis and will be presented in a future publication.

### Patients and treatments

The target population involved patients treated at the National Cancer Institute of Colombia (INC-ESE) diagnosed with advanced thyroid cancer who received treatment with sorafenib 800 mg/day (400 mg every 12 hours) up to a maximum of 24 months or until the occurrence of limiting toxicity, demonstrated disease progression, or voluntary withdrawal. All patients provided written informed consent before enrollment. Accrual was done sequentially after verification of inclusion criteria. These were as follows: adult patients >18 years old with confirmed histological diagnosis of differentiated thyroid cancer with inoperable locoregional or metastatic iodine-refractory involvement, which was defined as ( 8 ) a target lesion that does not show uptake in a gammagraphic evaluation after a given therapeutic dose under adequate conditions of elevated TSH and a low iodine diet; lesions that, despite showing iodine uptake, show progression after a dose of 100 mCi given in the previous 16 months; and patients showing progression after a cumulative dose greater than 600 mCi. All participants must have had tumor progression in the 12 months before enrollment, as suggested by several reports ( 9 , 10 ) and confirmed by the Response Evaluation Criteria in Solid Tumors, (RECIST version 1.1) ( 11 ), exhibit adequate functional status based on the Eastern Cooperative Oncology Group (ECOG) performance status (ECOG ≤ 2), and have a life expectancy of greater than 3 months. Previous use of chemotherapy, immunotherapy, or radiotherapy on the target lesion was permitted if the last dose was administered 4 weeks before study entry. Patients with uncontrolled cardiovascular diseases, unhealed wounds or ulcers, history of major bleeding in the 12 weeks prior to recruitment, thrombotic events in the last 6 months, or major surgery in the previous 4 weeks were excluded.

### Follow-up

Patients were evaluated at baseline, after the first month, and then quarterly with physical examinations, electrocardiograms, and laboratory tests. Imaginological evaluations were performed using chest tomography and neck ultrasound at Months 0, 3, 6, 12, 18, and 24 to evaluate treatment response (RECIST version 1.1 solid tumor response criteria ( 11 )). To evaluate toxicity, the classification system used in version 4.0 of the Common Terminology Criteria for Adverse Events (CTCAE) was used ( 12 ). Based on previous studies and the manufacturer's brochure, 4 levels of sorafenib dose reduction were used according to the severity of adverse events (Level 0: 800 mg per day, Level 1: 600 mg per day, Level 2: 400 mg per day, and Level 3: 200 mg per day).

### Statistical analysis

For statistical analysis, descriptive parametric and non-parametric statistics were included as appropriate. In the case of the qualitative variables, McNemar's test was used. For quantitative variables, paired Student's t-test and Wilcoxon Rank Sum tests were used for normally and non-normally distributed data, respectively. The Kaplan-Meier method was chosen to describe PFS and OS. Correlation analyses were performed between some markers and clinical variables and the observed outcomes using parametric and non-parametric coefficients.

This study was approved by the institutional research and ethics committee and was registered at Clinicaltrials.gov with the number NCT02084732 on March 12, 2014. Clinical monitoring as defined by the institutional research guidelines was conducted.

## RESULTS

**Figure 1:**
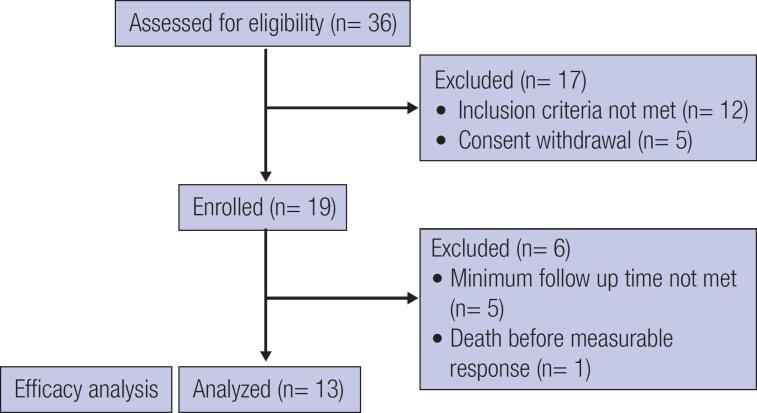
Patient flow diagram according to CONSORT guidelines.

Between August 2014 and November 2016, 36 patients were screened. Of these, 12 were not eligible, and 5 withdrew their consent. In the end, 19 patients were included in the study. However, for the efficacy analysis, 6 patients were excluded because they received only one month of therapy. Figure 1 presents patient flow according to the CONSORT guidelines. Thirteen (68%) patients were women, and the mean age at diagnosis was 61.8 years (range 38-84). All patients had metastatic lung involvement, 3 patients (15.7%) had additional bone involvement, and 10 patients (52.6%) had inoperable cervical metastases. All patients were subjected to locoregional resection at some time during the disease course. Baseline characteristics and prior treatments are summarized in Table 1 . Time from diagnosis to inclusion in the study was calculated with a median of 7.1 years (range 1.8-25.4).

**Table 1:** Baseline Clinical characteristics of the included patients

Variable	Disaggregation	Number	Percentage
Sex	Male	6	31.6
	Female	13	68.4
ECOG *	0	13	68.4
	1	5	23.3
	2	1	5.3
Comorbidities	Cardiovascular	14	73.7
	Articular	2	10.5
	Others	3	15.8
Thyroid cancer subtype	Papillary	16	84.2
	Folicullar	2	10.5
	Hürthle cell	1	5.3
Non operable cervical compromise	Yes	10	52.3
	No	9	47.7
Local radio-therapy	Yes	4	21.1
	No	15	78.9
I-131 doses (Cumulate)	Median (rank)	419	(160-950)

* Eastern Cooperative Oncology Group (ECOG) performance status.

**Figure 2:**
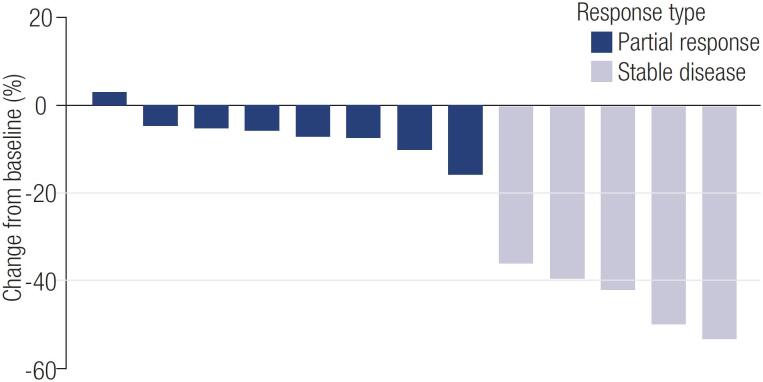
Best tumor response, RECIST criteria.

Median treatment time was 9.5 months (range 0.9-24.7). Twelve patients (63.1%) received sorafenib for more than 6 months, and 9 patients (47.3%) received it for at least 12 months. Tumor response could be evaluated in 13 patients. No complete responses were observed, but 5 patients experienced a partial response (35.7%) with a mean duration of 10.8 months on average. Furthermore, 6 patients had stable disease, and 3 showed progression. The best observed tumor responses are presented in Figure 2 .

**Figure 3:**
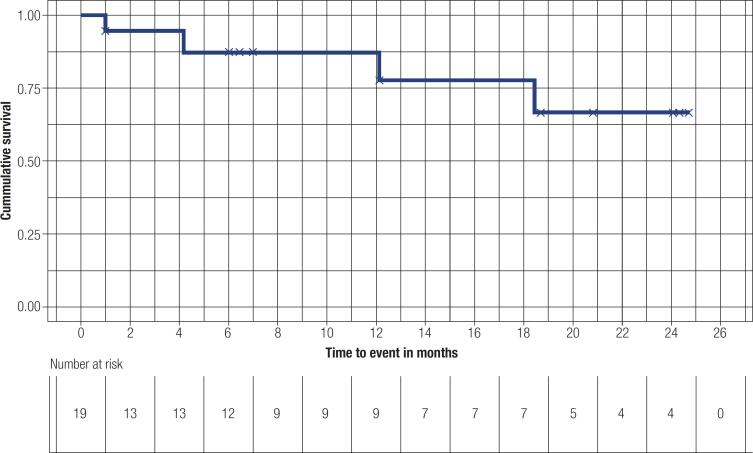
Progression free survival.

**Figure 4:**
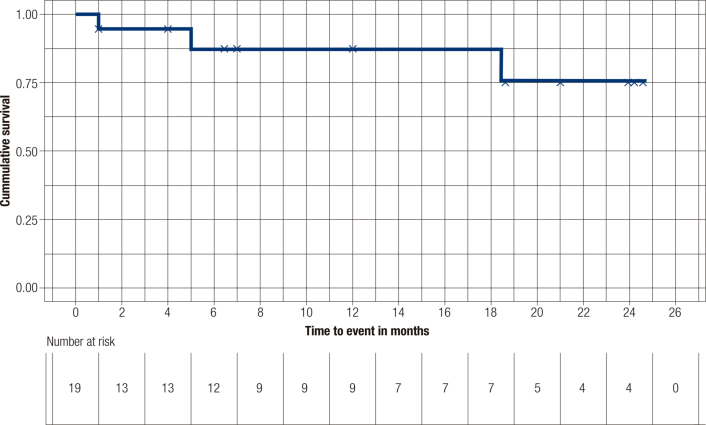
Overall survival.

Concerning PFS and OS, median values were not achieved for the cohort. An estimated mean PFS was calculated at 18 months (95% CI 10.7-20.3), whereas mean OS reached 21.3 months (95% CI 17.8-24.8). Survival curves are presented in Figures 3 and 4 , respectively.

Total events: 3; Mean progression-free survival: 18 months (95% CI 10.7-20.3).

Mean overall survival estimated at 21.3 months (95% CI 17.8-24.8).

With regard to thyroglobulin levels, no statistically significant differences (p = 0.075) were found between median values prior to treatment (1,223 ng/mL 95% CI 461-985) and the lowest achieved in the follow-up of patients receiving sorafenib (934 ng/mL 95% CI 473-1,394).

The most frequent adverse event was arterial hypertension, which occurred in all patients, followed by palmar-plantar erythema, which occurred in 13 of 19 patients (68.4%), and diarrhea, which occurred in 11 patients (57.8%). No deaths attributable to medication toxicity were documented. Table 2 summarizes adverse events by frequency and severity. Dose reductions and interruptions due to adverse events occurred in 4 (21.0%) and 7 (36.8%) patients, respectively. Palmar-plantar erythema and arterial hypertension were the most common reasons for sorafenib interruption. Dose withdrawals occurred in 3 (15.7%) patients, all of them between Days 15 and 21 of treatment. One experienced a Grade 3 skin rash, and the other 2 experienced Grade 3 hand-foot erythema. Despite resolution of symptoms, these patients withdrew consent to participate in the study. Grade 3 adverse events occurred in 8 (42.1%) patients, mainly hypertension and hand-foot erythema.

**Table 2:** Adverse events

Type	Description	Grade 1-2 n (%)	Grade 3 n (%)	Grade 4 n (%)
Dermatologic	Rash	4 (21.0)	3 (15.7)	0
	Stomatitis	4 (21.0)	1 (5.2)	0
	Hand/food Erythema	7 (36.8)	6 (31.5)	0
	Alopecia	1 (5.2)	0	0
General symptoms	Muscle pain	4 (21.0)	1 (5.2)	0
	Fever	1 (5.2)	0	0
	Weight loss	3 (15.7)	0	0
Gastro-intestinal	Diarrhea	6 (31.5)	4 (21.0)	1 (5.2)
	Nausea/vomiting	3 (15.7)	1 (5.2)	0
	Dysphagia	1 (5.2)	0	0
	ALT/AST elevation	1 (5.2)	0	0
	Cholangitis (biliary tract infection)	0	1 (5.2)	0
Pulmonary	Dyspnea/cough	0	1 (5.2)	0
Cardiovascular	Arterial hypertension	8 (42.1)	7 (36.8)	4 (21.0)
	Tachycardia	1 (5.2)	0	0
	Acute myocardial infarction	0	1 (5.2)	0
Other	Creatinine elevation	0	1 (5.2)	0

A total of 3 patients (15.7%) died during the study period. The first patient experienced severe abdominal pain (10/10) after 20 days of treatment. Following this event, sudden death was declared. Due to non-consent on the part of the family to conduct an autopsy, specific organ alteration could not be confirmed. With suspicion of a ruptured abdominal aortic aneurysm, this event was reported to the regulatory agency overseeing the trial. Because no specific cause of death could be confirmed, it was not included in Table 2 . Another patient had a serious adverse event (myocardial infarction) at 6 months of treatment; medication was discontinued, and the patient subsequently died of tumor progression 3 months after withdrawal. The final patient presented with disease progression under treatment at 18 months and died after one month of sorafenib withdrawal.

## DISCUSSION

The frequency of distant metastases at diagnosis in differentiated thyroid carcinoma varies according to the histological type. It is estimated at approximately 10% in papillary carcinomas, 25% in follicular carcinoma, and up to 35% in Hürtle cell carcinoma ( 13 – 15 ). Pulmonary metastatic involvement is the most frequently involved site and was present in 100% of the patients in the current study. In addition, the presence of coexisting regional lymph node involvement should be kept in mind, given the morbidity that it entails. In the present study's population, 52% of the patients had inoperable cervical involvement, a figure that contrasts with the presence of local involvement in the DECISION trial of only 3.4% ( 4 ).

The 10-year survival prognosis in patients with metastases varies according to the age at the time of diagnosis; it is 95% in patients younger than 45 years, but it is reduced to 50% in patients older than 45 years ( 14 , 15 ). In this study, the proportion of reported deaths (15.7%) was greater than the 5% of deaths reported in the DECISION Trial ( 4 ), and the 3.3% reported by Gupta-Abramson and cols. ( 16 ) but less than the 42% of deaths reported by Kloos and cols. ( 17 ). Bearing in mind that disease progression was the major cause of death, is important to mention that in the DECISION trial, 20.3% of patients randomized to sorafenib received systematic treatments after disease progression, unlike in this study, mainly because of the absence of the availability of other therapies. Another difference between the population included in both studies was the median time from diagnosis to inclusion, which was slightly longer in the present study (84 months, range 21-300) than in the DECISION trial (66 months, range 3.9-362).

The partial response (PR) rate was higher (35.7%) compared to observed in previously published phase II studies by Gupta-Abramson and cols. ( 16 ) and Kloos and cols. ( 17 ), which had PR rates of 28 and 15% respectively. It was also higher than the DECISION trial's PR rate of 12%. In the same way, the median PFS observed in our study (18 months) was longer than in the DECISION TRIAL (10.4 months) but similar to the PFS found by Gupta-Abramson and cols. (19.7 months) and by Kloos and cols. (16 months).

It corroborates the benefit of sorafenib in patients in whom the disease progresses and who lack other therapeutic options. Considering the very small percentage of Hispanic patients included in the DECISION trial (∼1%), without being able to compute statistical comparisons, it is possible that tumor behavior could have differed in our population, thus warranting our conducting this phase II study in local and Hispanic populations. Furthermore, considering the benefit of prolonged median PFS for patients harboring somatic BRAF V600E mutations compared to their BRAF wild type counterparts (20.5 versus 8.9 months respectively), it is safe to assume that populations with higher mutational incidence can benefit from treatment with sorafenib to a greater extent. In addition, the prevalence of BRAF V600E mutations in DTC patients from Colombia has been reported to be between 60% and 66%, which is higher than reported in the DECISION trial (30%) ( 18 , 19 ). This might explain the high response rates observed in this study.

The most frequent serious side effects in this study were different than those reported in other studies ( 4 , 16 , 17 ) and involved the cardiovascular system, with arterial hypertension in 4 patients (21%) and acute myocardial infarction in 1 (5.2%). It is important to mention that 14 (73.7%) patients in this study had cardiovascular comorbidities. In addition, hypertension as an adverse event of antiangiogenic agents can be a marker of effective target inhibition, a greater response rate, PFS, or even OS ( 20 ), potentially explaining these best-observed responses.

Although there is increasing evidence of the benefit of Lenvatinib use, its safety profile differs, probably due to a greater antiangiogenic effect. Moreover, there are some rare but potentially fatal adverse events reported with its use, such as tracheoesophageal fistula, gastrointestinal perforation, and reversible rear leukoencephalopathy syndrome ( 21 ). It is therefore pertinent to consider sorafenib as an alternative therapy in cases of possible contraindications, such as patients with proteinuria or high risk of serious adverse events ( 22 , 23 ).

As more has become known about the limitations of radioactive iodine in differentiated thyroid carcinoma ( 24 ), the use of tyrosine kinase inhibitors has increased for patients with advanced disease in progression, who would have been considered intractable before these molecules were available.

In conclusion, the present study demonstrates the benefit of sorafenib for palliative use in Colombian patients with advanced progressive DTC who lack any other therapeutic options. Results in the setting of advanced thyroid carcinoma refractory to radioactive iodine are comparable in efficacy and safety, similarly to previous conclusions of other studies.
